# The study of a hermaphroditic sheep caused by a mutation in the promoter of *SRY* gene

**DOI:** 10.1016/j.vas.2023.100308

**Published:** 2023-07-24

**Authors:** Farid Heidari, Mohaddeseh Rahbaran, Asieh Mirzaei, Mehran Mozafari Tabatabaei, Sara Shokrpoor, Frouzandeh Mahjoubi, Mehdi Shams Ara, Vahid Akbarinejad, Faramarz Gharagozloo

**Affiliations:** a Department of Animal Biotechnology, Faculty of Agriculture Biotechnology, National Institute of Genetic Engineering and Biotechnology (NIGEB), Tehran, Tehran, Iran; b Department of Animal Sciences, Shahid Bahonar University of Kerman, Kerman, Kerman, Iran; c Department of Pathology, Faculty of Veterinary Medicine, University of Tehran, Tehran, Tehran, Iran; d Department of Medical Genetic, Institute of Medical Biotechnology, National Institute of Genetic Engineering and Biotechnology (NIGEB), Tehran, Tehran, Iran; e Department of Theriogenology, Faculty of Veterinary Medicine, University of Tehran, Tehran, Tehran, Iran

**Keywords:** Disorders sex developments, Mutation, Shal sheep, *SRY* gene

## Abstract

In mammals, sex-determining region Y (*SRY*) gene plays vital role as a transcription factor to regulate the expression of the genes contributing to development of male genitals. Any mutation disrupting expression of *SRY* gene can cause disorders of sex development (DSDs).

In this study, the examination of a hermaphroditic (female-like) Shal sheep which was referred for infertility is described. Initially, the reproductive system of the sheep was histologically and anatomically assessed. Karyotyping was used to determine the real gender of the animal. Sex hormones including progesterone, estradiol, and testosterone were measured by enzyme-linked immunosorbent assay (ELISA). Eventually, promoter part and *SRY* gene were sequenced and aligned to detect any potential mutation using NCBI data base.

Although anatomical inspection led to identification of uterus, ovary, and enlarged clitoris as well as testes in the sheep, the karyotyping results interestingly revealed that the animal was genetically a male. Although the sheep had both male and female gonads, there were no overt signs of reproductive behavior and gamete production was not observed. Plasma steroid hormone levels were reported to be at basal levels. Additionally, a mutation was detected on the promoter of the SRY gene.

In conclusion, the case implies that mutation on the promoter part of *SRY* gene could disrupt sexual development of the fetus culminating in DSDs in the sheep.

## Highlights

• The *SRY* gene has a key role in sex development.
• In the absence of SRY protein, the femal genital system is developed.
• Genetics defects are the major cause of disorders of sex development (DSDs) in mammals.
• Mutation in SRY promoter may result in DSDs.

## 1 Introduction

The SRY gene, located on the Y chromosome in mammals, encodes the sex-determining region Y (SRY) protein, also known as testis-determining factor (TDF). This protein is one of the SOX (SRY-like box) transcription factors responsible for the development of gonadal cords, which subsequently differentiate into seminiferous tubules (Olivares et al., 2018; Polanco & Koopman, 2007). Obviously, any mutation on coding or regulatory regions of the SRY gene can disrupt the development of the male genitals. The disruption is known as DSDs which appears as various sexual phenotypes. DSDs include a group of congenital conditions with unusual development of genital structures (MAatouk et al., 2008; Witchel, 2018). In the DSDs cases, atypical external genitalia might be present at birth or recognized later by delayed/absent puberty, postnatal virilization or infertility (Wisniewski et al., 2019). Although presence or absence of Y chromosome usually determines the phenotypic gender of an individual, there are some rare cases in which the karyotype and phenotypic gender does not match (Witchel, 2018). This disturbance in sexual development might not be limited to reproductive organs and would lead to observation of aberrant sexual behaviors (Kyriakou et al., 2015). This issue could stem from three conditions including establishment of chromosomal sex, formation of gonadal sex, and development of phenotypic sex, which play crucial roles to shape the sexual identity of an individual, either physically or mentally (Edson et al., 2009; Villagomez et al., 2009). Any defects in the corresponding developmental events can cause disorders of sex development (DSDs) which would be accompanied by sexual indeterminateness and dysfunction of reproductive organs. In this regard, the proper expression of the genes that regulating formation of sexual phenotype could be really important, and it is worth noting that upregulation of genes associated with male gender results in development of male sexual organs formation and male behaviors, whereas absence or silence of these genes would lead to development of female sexual organs and behaviors (Ahmed & Hughes, 2002).

Indeed, the term DSD has a general definition including any type of developmental abnormality in which the phenotypic gender and karyotype does not match (Hughes et al., 2006; Kun & Jongwon, 2012). Although DSDs have been reported in human as well as farm animals, data associated with incidence of DSDs indicate that these conditions are rare and the total occurrence of DSDs is estimated at 1 in 5500 in animal (Bashamboo & McElreavey, 2016; Sax, 2002). In this context, hermaphroditism has been reported in ewe (Smith et al., 1996; Fayrer-Hosken et al., 1992), goat (Pailhoux et al., 2001)) and cattle (Bresciani et al., 2015). Yet there is limited information about DSDs in wild species (Birk et al., 2000; Mastromonaco et al., 2012).

Disorders of sex development are classified into three categories including DSD sex chromosomes, DSD 46, XX, and DSD 46, XY (Houk & Lee, 2008). With respect to diagnosis of DSDs, a number of studies have concentrated on the identification of genetic variants causing the unusual sexual development by various techniques (Shen at al., 1994). Sequencing, as an effective technique to analyze duplication and deletion in DNA, has been applied in approximately 50% of cases to identify the underlying cause for the condition (Eggers et al., 2016; Kyriakou et al., 2015).

Sex-determining region Y (*SRY*) gene plays a key role in sexual development and is responsible for determining male sex. Therefore, any mutations conducted in *SRY* could culminate in a wide range of DSDs in the fetus. *SRY* transcription factor (TF), known as testis-determining factor (TDF), is actually a DNA-binding protein regulating testicular formation and development (García-Acero et al., 2020). *SRY* protein can enter the nucleus and binds to the specified cis-elements on promoters of the genes encoding other TFs including SOX9, CBLN4, and ER7 1/ ETV2, which are involved in differentiation and proliferation of Sertoli cells and the tubular structures of the testes (Arboleda et al., 2014). Hence, pathogenic mutation of the *SRY* gene is able to sexually reverse male to female or stimulate simultaneous creation of both testicular and ovarian tissue (McClelland et al., 2012). In corroboration of this notion, mutation specially in SRY gene has been recognized to be responsible for hermaphroditism in goat and cattle (Pailhoux et al., 2001; Bresciani et al., 2015).

In the present study, a hermaphroditic sheep which was referred to veterinary hospital for a history of infertility was investigated anatomically, physiologically and molecularly.

## 2 Materials and methods

### 2.1 Clinical/case data

A healthy 2.5-year-old Shal sheep, which was apparently-female, was referred to veterinary hospital with a history of infertility. The weight of the sheep was 38 kg at the time of examination. The sheep under investigation did not have a history of estrus, mating, pregnancy, or parturition, while the other female members of the herd were fertile and had a record of successful pregnancy and parturition. The herd was managed using a pasture-based and grazing system.

### 2.2 Karyotyping

Cytogenetic analysis of phytohemagglutinin (PHA)-stimulated peripheral blood leukocytes was performed in accordance with a standard protocol. In brief, blood lymphocytes were cultured in 5 mL RPMI 1640 (Gibco®; Invitrogen, Paisley, Scotland, UK) supplemented with 20% (v/v) fetal bovine serum (GIBCO®; Invitrogen) and 10 μL/mL phytohemagglutinin (PHA) (GIBCO®; Invitrogen) at 37 °C. After 68 h of incubation, 50 μL colcemid (10 μg/mL; GIBCO®; Invitrogen) was added to the cultured cells, which were incubated at 37 °C for about 12 min. Afterwards, the cell suspension was centrifuged, and the pellet was resuspended in 5–10 mL KCL (0.075 M) for about 20 min at 37 °C. Following the centrifugation, the cells resuspended in fixative (3v methanol l:1 acetic acid; Merk, Frankfurt, Germany), and the fixative was changed at least three times. Using a Pasteur pipette, a drop of cell suspension was placed onto the slide. The chromosomes were viewed under phase contrast microscope to assess the quality of the metaphases and nuclei. When aging was over, the chromosomes were treated with trypsin, then stained with Giemsa (GTG-banded).

### 2.3 Genomic analyses

#### 2.3.1 DNA sequencing

Initially, genomic DNA was extracted from blood cells collected in an EDTA collection tube by DynaBio™ Blood/Tissue DNA Extraction Kit (Takapo Zist Co., Tehran, Iran). We used the Primer-BLAST tool available at https://www.ncbi.nlm.nih.gov/tools/ primer-blast/ to design specific forward (5´-CTAATTGGTCCTTGTCTCTGC-3´) and reverse (5´-CATCTTACTGTGGATGCAAACG-3´) primers based on the conserved sequences of SRY gene promoter region of Ovis aries (HQ840956). PCR reaction was carried out in a total volume of 25 μL including 12.5 μL 2X Taq master mix (Kiagene Fanavar cat. No. FPLF007.1000) plus 5 pmol of each primer and 100 ng of genomic DNA as template. The amplification was carried out under the thermal cycles of 95 °C for 5 min, 30 cycles of 95 °C for 30 s, 52 °C for 30 s and 72 °C for 45 s. A final extension at 72 °C for 10 min was included. The PCR product was transferred to Takapo Zist Co., Tehran, Iran, for sequencing the pro*SRY* gene by specifically designed primers.

#### 2.3.2 Multiple alignments

The *SRY* region (Include promoter region) were amplified and sequenced using Sanger method. The ABI files were opened by the Finch TV software and aligned with the relevant sequences obtained from NCBI using the Megalign program by the ClustalW method in the DNASTAR software package.

### 2.4 Assessment of steroid hormones

Jugular vein blood sample was collected using Venoject tubes containing heparin sodium. The levels of progesterone (MyBiosource cat. No. MBS 703,845, intra-assay CV ≤ 15%; inter-assay CV ≤ 15%), estradiol (MyBiosource cat. No. MBS 702,098, intra-assay CV ≤ 15%; inter-assay CV ≤ 15%), and testosterone (MyBiosource cat. No. MBS 701,270, intra-assay CV ≤ 15%; inter-assay CV ≤ 15%) in the plasma were measured using enzyme-linked immunosorbent assay (ELISA). Each hormone assay was performed in triplicate.

### 2.5 Necropsy and histological assay

Samples were taken from the reproductive tract and fixed in 10% neutral buffered formalin, routinely processed, dehydrated and embedded in paraffin wax, sectioned at 5 μm in thickness (Rotary Microtome RM2 145; Leica, Germany) and stained with hematoxylin-eosin (H&E). Sections were examined by a light microscope (E600; Nikon) and representative images were taken.

## 3 Results

### 3.1 Karyotype

**Fig. 1:**
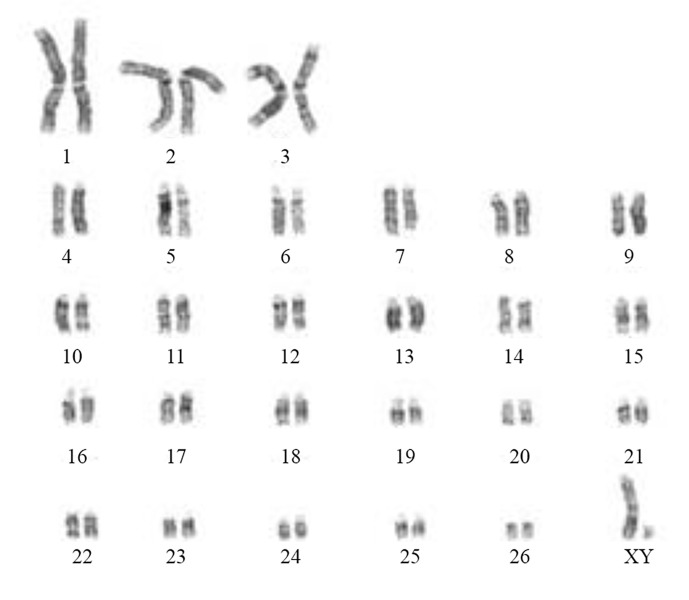
The karyotype of the studied sheep.

Karyotyping showed 27 chromosome pairs (2n = 54) with XY as the sex chromosome pair as shown in Fig. 1. Thus, the sheep was chromosomally male.

### 3.2 Nucleotide substitution in SRY promoter

**Fig. 2:**
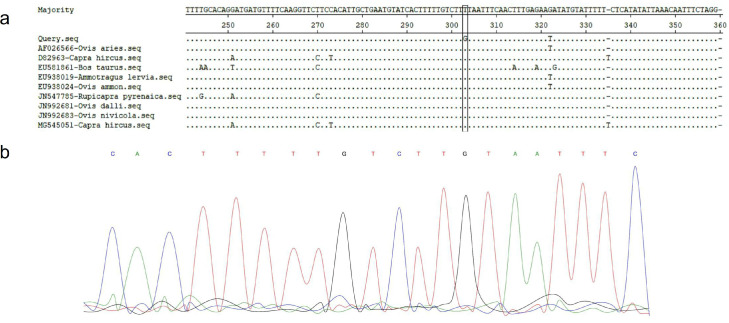
(a) The variation site (nucleotide No.303) in the SRY promoter, (b) The sequencing chromatogram of the variation site in the SRY promoter.

In Fig. 2-b, the sequencing chromatogram is displayed. The study involved alignment and comparison of the SRY promoter sequences of the intersexual sheep to those of Ovis aries and other relevant species. As shown in Fig. 2-a, a single nucleotide substitution (T to G) was detected in the promoter region of the SRY gene (nucleotide number 303) in the hermaphrodite sheep.

### 3.3 Steroid hormones analysis

The mean levels of progesterone, estradiol, and testosterone were 0.08 ng/mL, 20.2 pg/mL, and 0.1 ng/mL, respectively.

### 3.4 Post mortem examinations of the urogenital system

**Fig. 3:**
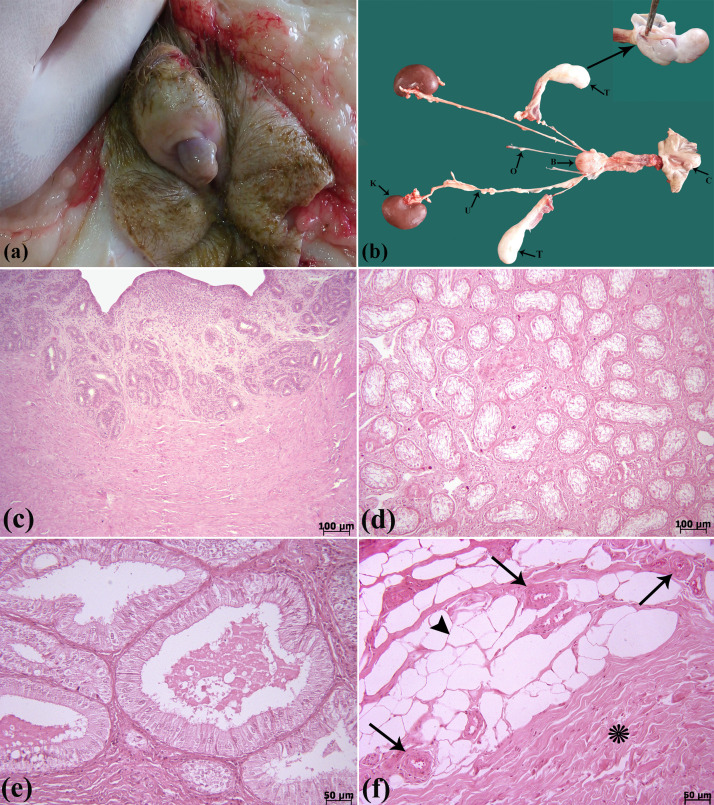
**(a–f)** Macroscopical and microscopical findings of DSD. **(a):** Vulvar meatus with projection of a large clitoris resembling a penis. (**b**): Gross findings of urogenital organs, K (kidney), U (ureter), B (urinary bladder), O (ovary), T (testis) and C (clitoris), inset: (testis). (**c**): Histopathological sections of uterus, **(d):** testis, **(e):** ductus epididymidis, **(f):** ovary, blood vessels (arrows), lipocytes (arrowhead) and connective tissue (*), H&E.

At gross examination, an enlarged clitoris resembling a penis was observed. Investigation of internal organs revealed the ovaries, uterus (body and horns) and bilateral cryptorchid testes. The testes, much smaller than in adult males, were connected to epididymis and deferent ducts. Additionally, ovaries and oviducts were found on both sides (Fig 3a-b).

### 3.5 Microscopic examination of the urogenital system

Histological examination of uterus revealed the surface epithelium of endometrium was simple columnar. Simple coiled and branched tubular glands were present throughout the endometrium. The myometrium and perimetrium layers were also detected (Fig 3c). Microscopically, the seminiferous tubules did not show the evident ability of spermatogenesis in the testis. In this context, few cells were observed in the basal epithelium of seminiferous tubules. In addition, spermatocytes and spermatids were absent in the seminiferous tubules (Fig 3d). The epididymis was lined by a pseudostratified columnar epithelium, surrounded by a small amount of loose connective tissue and circular smooth muscle fibers (Fig 3e). Microscopically, blood vessels, lipocytes and connective tissue with collagen fibers instead of normal ovary structures such as various stages of follicles were observed in the ovary tissues (Fig 3f).

## 4 Discussion

Shal is a local variety of fat-tailed sheep, which is commonly raised at pastures in the village of Shal, Buin Zahra city, Qazvin province, Iran and considered as meat type breed (Akbarinejad et al., 2014; Eghbalsaied et al., 2017). In this study, a female-like 2.5-year-old Shal sheep was molecularly, hormonally and histologically analyzed. She was clinically healthy and fed on pasture. There was no history of the estrus, mating, fertility or pregnancy. Other ewes of this herd were clinically and reproductively normal.

Generally, Sheep (*Ovis aries*) is a diploid organism with chromosome no. (2n = 54) including XX and XY chromosomes in female and male respectively (Eghbalsaied et al., 2017). According to the karyotyping results, the case was genetically a male (XY). But, the results of necropsy showed that it has female genital system (vulva, vagina, cervix, uterus, oviduct and ovary shape mass without any reproductive function and follicular formation) in addition to small and non-developed testes and an enlarged clitoris. No sign of spermatogenesis, spermatocytes and spermatids were observed in the seminiferous tubules. The results of DNA sequencing indicated that there was a mutation in the promoter of *SRY* gene. It is well established that male phenotype is strictly correlated with the presence of the Y chromosome, and the number of copies of the X chromosome does not matter. The only exception to this rule occurs when mutations or deletions in genes of the sex determination pathway impair the function of the Y chromosome (Pask & Graves, 2001). In humans, various mutations have been reported in the sex determination pathway gene, both in the coding regions (Achermann et al., 1999; Umehara et al., 2000; Biason-Lauber et al., 2009; Fluck et al., 2011; Pearlman et al., 2010; Portnoi et al., 2018) and upstream regions (Zhou et al., 2021), in association with cases of DSD. Unfortunately, information about the effects of gene mutations in DSDs in animals is limited. De Lorenzi et al. (2018) reported a DSD in cat with female presentation and activation and XY chromosome. In 2018, Lorenzi et al. reported a case of DSD in a cow with XY chromosomes, but exhibiting a female phenotype. Scholz et al. reported a case of a hermaphrodite cat in 2010, where no mutations were found in the coding region of the SRY gene. In our study, we evaluated both the coding and regulatory regions of the gene, and we identified a mutation in the regulatory region. In some previous study, focusing solely on the coding region of a gene may result in the failure to identify mutations. In 2020, Guang-Xin et al. reported a novel complex PIS variant in the genome, which serves as a broad-spectrum clinical diagnostic marker for XX intersexuality in goats from Europe and China.

In human, Swyer syndrome is a DSD that is characterized by the failure of sexual development. In these cases, sexual development does not match the affected individual's chromosomal set (King & Conway, 2014). The incidence of Swyer syndrome is 1 in 80,000 individuals (Jabeen et al., 2019). Mutation in genes has been indicated as the major cause of Swyer syndrome and more than 70% of reported mutations were located on *SRY* gene (Chand et al., 2020).

Sex chromosome abnormalities are generally better tolerated by animal species; however, some genes escape from inactivation and cause reproductive disorders leading to abnormal development of internal sex organs (Burgoyne et al., 2002). Sex reversal syndrome occurs when male and female phenotypes (or gonadic sex) differ from the expected sex chromosome constitution, as in XX males and XY females (Iannuzzi et al., 2021). This syndrome has been reported in all domestic bovids (Cattle, Buffalo, Sheep and Goat). Two cases were reported with sex reversal syndrome in sheep where 54 XY sheep showed female phenotype. The mutation in *SRY* gene was not reported in these cases (Albarella et al., 2019; Ferrer et al., 2009). Two cases of XY sex reversal syndrome have been observed in river buffalo, which were sterile with severe disruption in their internal sex organs. However, upon investigation by FISH-mapping and gene-sequence analysis, one individual displayed the *SRY* gene at the expected location on Y chromosome with normal DNA sequence (Patel et al., 2006).

## 5 Conclusion

In conclusion, *SRY* or TDF protein is the vital player for normal development male genitals and organs. A mutation in the regulatory region of the *SRY* gene could led to malformation of male genital system. In this case, a genetically male Shal sheep with female appearance and organs with history of infertility was described. The sheep had small testes with no evident spermatogenesis. Indeed, this case implicates the *SRY* gene plays a crucial role in normal development of male reproductive organs in sheep and mutations of SRY could results in DSD leading to infertility.

## Statement of ethics

Behalf of all authors, I want to confirm that all experiments on the sheep were carried out under directive 2010/63/EU that approved and used by “Ethics committee of National Institute of Genetic Engineering and Biotechnology” (IR.NIGEB.EC.1394.8.10).

## Declaration of Competing Interest

The authors declare that they have no known competing financial interests or personal relationships that could have appeared to influence the work reported in this paper.
